# Seaweed and melatonin in the induction of tolerance to recurrent water deficit in papaya

**DOI:** 10.3389/fpls.2025.1630028

**Published:** 2025-10-01

**Authors:** Thayanne Rangel Ferreira, Giuseppe Tognere Polonini, Letícia Freitas Fonseca, Cristhiane Tatagiba Franco Brandão, Antelmo Ralph Falqueto, Edilson Romais Schmildt, Vinicius de Souza Oliveira, Lúcio de Oliveira Arantes, Enilton Nascimento de Santana, Sara Dousseau-Arantes

**Affiliations:** ^1^ Departamento de Ciências Biológicas, Centro de Ciências Humanas e Naturais, Universidade Federal do Espírito Santo, Vitória, Espírito Santo, Brazil; ^2^ Centro de Pesquisa, Desenvolvimento e Inovação Norte, Instituto Capixaba de Pesquisa, Assistência Técnica e Extensão Rural, Linhares, Brazil; ^3^ Departamento de Ciências Agrárias e Biológicas, Centro Universitário Norte do Espírito Santo, Universidade Federal do Espírito Santo, São Mateus, Espírito Santo, Brazil

**Keywords:** biostimulants, *Carica papaya* L., drought cycles, chlorophyll a fluorescence, growth regulator

## Abstract

**Introduction:**

Abiotic stresses cause physiological and biochemical imbalances, such as stomatal closure, reduced photosynthesis, and changes in water balance, biomass allocation, and carbohydrate metabolism, compromising growth and, consequently, productivity. One strategy to minimize the effects of these stresses in agriculture is the use of biostimulants. Therefore, the objective of this study was to evaluate the effects of foliar applications of melatonin, *Ascophyllum nodosum*, and *Lithothamnium calcareum* on papaya plants subjected to three recurring cycles of water deficit on physiological performance, carbohydrate allocation, and vegetative growth.

**Methods:**

Three water deficit and recovery trials were conducted on ‘Aliança’ papaya seedlings. Before imposing the water deficit, solutions of the biological regulator melatonin and seaweed extracts from *A. nodosum* and *L. calcareum* were applied via foliar application. Water potential, chlorophyll a fluorescence, photosynthetic pigments and vegetative growth of seedlings were evaluated.

**Results:**

Seaweed and melatonin promoted increased water retention by decreasing leaf water potential and maintaining and restoring photosynthetic functions. In the second cycle of water deficit, there were significant reductions in maximum photochemical quantum yield and an increase in the energy flux dissipated per reaction center. Seaweed and melatonin also reduced total soluble sugar levels. Melatonin also promoted increased growth in specific stem length and specific root length.

**Discussion:**

Foliar applications of melatonin, *A. nodosum*, and *L. calcareum* proved effective in mitigating the effects of water deficit in papaya seedlings. Chlorophyll a fluorescence indicated that photosynthetic functionality was most affected during the second drought cycle, with inhibition of the photosynthetic apparatus during this period. Water stress reduced chlorophyll levels, possibly as a strategy to minimize photooxidative damage. Among the biostimulants tested, melatonin stood out in terms of specific stem length and specific root length growth, indicating greater adaptation to water deficit.

## 1 Introduction

The papaya tree (*Carica papaya* L.) is one of the most widely planted and consumed tropical fruits in the world. In 2023, approximately 1,423,583 tons of papayas were produced worldwide. Brazil stands out as the fifth largest producer of papayas in the world, with 1,138,343 tons (FAO, 2025). Furthermore, in 2023, the state of Espírito Santo, the target region of the study, ranked second in Brazil, with 352,046 tons of papayas produced, corresponding to approximately 31% of Brazilian production (IBGE, 2025). Papayas produced in Espírito Santo are known for their excellence and quality, and are exported to the European Union and the United States of America, mainly by air (Zucoloto et al., 2023).

The increase in the frequency and intensity of water scarcity is a global concern for agriculture, and this situation is leading to changes in the production systems of all crops, including papaya (Rahimi et al., 2022). Especially in semiarid regions in Brazil where the crop is most produced (Souza et al., 2022), water scarcity causes detrimental effects on the physiological, biochemical processes and growth of papaya (Mahouachi et al., 2023). Under water scarcity conditions, papaya reduces net CO_2_ assimilation due to stomatal closure (Ruas et al., 2022). Although the photosystems continue to absorb photons, the captured energy cannot be efficiently used in carbon reduction (Ruas et al., 2022). Stomatal closure reduces transpiration, resulting in increased leaf temperature, which deactivates Rubisco, reducing its efficiency and limiting CO_2_ fixation (Fathi and Tari, 2016). This effect compromises both vegetative growth and production.

A promising alternative is the use of biostimulants, these products have emerged as effective alternative tools to improve plant nutrition and increase the ability of plants to tolerate environmental stresses (Shukla and Prithiviraj, 2021). Biostimulants based on seaweed extract such as *Ascophyllum nodosum* contain a wide variety of substances, including phenolic compounds, alginic acid, mannitol, laminarin, betaines, proteins, lipids and several mineral nutrients in different concentrations (Shukla et al., 2019). Among red seaweeds, the genus *Lithothamnium* also has biostimulant potential, as its organic fraction is rich in humic acids with auxinic activity (Amatussi et al., 2023). In addition to seaweed, melatonin (N-acetyl-5-methoxytryptamine) is a biological regulator that modulates physiological and biochemical mechanisms, stimulating plant growth (Debnath et al., 2018). Biodegradable and non-toxic, its application is considered a sustainable alternative in agriculture (Janas and Posmyk, 2013).

Under water deficit conditions, applications of *A. nodosum* and melatonin were reported to contribute to the regulation of osmotic potential in tomato, grapevine, wheat, and potato plants (Goñi et al., 2018; Hossain et al., 2020; Frioni et al., 2021; El-Yazied et al., 2022). Hossain et al. (2020) observed, in wheat plants treated with melatonin under water deficit, increases in maximum quantum efficiency, root length, and root diameter. Goñi et al. (2018) highlighted that *A. nodosum* increased chlorophyll levels in tomato seedlings under water deficit. Increasing chlorophyll levels during and after water deficit may be beneficial for restoring the photosynthetic capabilities of leaves and, in combination with other metabolic processes, lead to growth recovery (Goñi et al., 2018). Maradiaga-Rodrigues et al. (2018) reported higher sucrose content in sugarcane (*Saccharum* sp.) with the use of *Lithothamnium* combined with vinasse, in dryland cultivation. In addition, Carvalho et al. (2019) reported improvements in photosynthetic parameters in grapevines (*Vitis vinifera* L.) under water deficit.

Despite the proven benefits of these bioinputs in several production systems, the literature still presents gaps regarding their effects on papaya crops, especially under recurrent water deficit. Although previous studies have indicated improvements in the quality of papaya seedlings with the use of *A. nodosum* and *Lithothamnium* (Guimarães et al., 2012; Ramos et al., 2023), there is a gap in the understanding of the physiological and biochemical effects. In addition, there is a lack of studies involving the use of melatonin in papaya crops under water deficit, highlighting the need for further investigations in this area. Thus, the objective of this study was to evaluate the effects of foliar applications of melatonin, *A. nodosum* and *L. calcareum* on ‘Aliança’ papaya trees subjected to recurring cycles of water deficit, on physiological performance, carbohydrate allocation and vegetative growth.

## 2 Materials and methods

### 2.1 Plant material and cultivation conditions

The experiment was conducted between November and December 2023, at the Linhares Experimental Farm, of the Capixaba Institute for Research, Technical Assistance and Rural Extension (Incaper), located at 19°25’0.1”S and 40°4’35.3”W, in the municipality of Linhares, northern region of the state of Espírito Santo. The seedlings were propagated via seed, using the adapted method described by Oliveira et al. (2013).

Three seeds of the papaya tree (*Carica papaya* L.) of the ‘Aliança’ cultivar were sown 2 cm deep in tubes with a capacity of 55 cm^3^. The tubes were filled with Tropstrato HT vegetable substrate, consisting of pine bark, vermiculite, PG Mix 14.16.18, potassium nitrate, simple superphosphate and peat. In addition, 1.5 g of Basacote^®^ Mini 3M 16-8-12 (+2) per tube was added to the substrate. The seedling propagation period was conducted in a nursery covered with a black shade cloth with 50% shading and irrigation carried out by means of micro sprinklers with a flow rate of 7 liters per hour (L h^–1^), for three minutes, activated every two hours.

After the emergence of the seedlings, thinning was carried out, keeping only the most vigorous seedling per tube. When the seedlings reached the commercial standard of 15 to 20 cm (Oliveira et al., 2013), they were transplanted into plastic pots with a capacity of 7 L. Transplanting was done using the adapted method described by Oliveira et al. (2013). The pots were filled with Tropstrato HT vegetable substrate and fertilized with 17 g of Basacote^®^ Mini 3M 16-8-12 (+2). After transplanting, the seedlings were transferred to a greenhouse with a polyethylene cover and 30% Aluminet screen, where they remained for 30 days for acclimatization, being irrigated regularly to maintain field capacity.

The treatments consisted of foliar applications of melatonin (100 µM), *Ascophyllum nodosum* (3 mL L^−1^), *Lithothamnium calcareum* (3 mL L^−1^) and distilled water (control). The doses were selected based on studies by Silva et al. (2012); Ye et al. (2016); Alagupalamuthirsolai et al. (2023); Ramos et al. (2023) and Ferreira et al. (2025), which demonstrated positive effects of these biostimulants in mitigating stress in plants under adverse conditions. The applications were made with a 5 L manual sprayer (model 5 L, Starfer brand). The control treatment was maintained at field capacity throughout the experiment.

The melatonin solution was prepared as described by Ye et al. (2016), by dissolving 0.69 g of melatonin (Sigma Aldrich) in 15 mL of ethyl alcohol as a stock solution. Subsequently, aliquots of 2.52 mL were removed from the stock solution to obtain the concentration of interest (100 µM) and were made up to 5 L with distilled water.

The 3 mL L^–1^ solution of *A. nodosum* was prepared by dissolving 15 mL of the commercial product Baltiko^®^ from Litho Plant in 5 L of distilled water. According to the manufacturer, Baltiko^®^ is composed of A. nodosum extract, amino acids, humic substances and water. The label does not specify the percentage of algae extract, humic substance or which amino acids are present in the product. The product guarantees 5% water-soluble potassium (63.0 g/L); 2% water-soluble nitrogen (25.20 g/L); 14% total organic carbon (176.40g/L); Water solubility at 20°C 100g/L; Electrical conductivity at 28.50 mS/cm; density of 1.26 kg/L; saline index of 23.50%; pH 7.57; highest solute/solvent ratio recommended by the manufacturer 100 g/L and is of a fluid and suspended physical nature.

To complement the knowledge about the nutritional effects of products based on A. nodosum, a sample of the product was sent to the Agronomic, Environmental Analysis and Preparation of Chemical Solutions laboratory for analysis of macro and micronutrients, results expressed in **Table 1**.

**Table 1:** Analysis of macro and micronutrients of the commercial product Baltiko^®^ based on *Ascophyllum nodosum*, including the following elements: nitrogen, phosphorus, potassium, calcium, magnesium, sulfur, iron, zinc, copper, manganese and boron.

Product/parameters	Baltiko^®^		
	Low	Medium	High
Total nitrogen [%m/m]			2.2
Total phosphorus [%m/m]	0.3		
Total potassium [%m/m]			5.7
Total calcium [%m/m]	0.1		
Total magnesium [%m/m]	0.03		
Sulfur [%m/m]			1.9
Iron [%m/m]	0.04		
Zinc [ppm]	0.27		
Copper [ppm]	0.07		
Manganese [ppm]	0.47		
Boron [ppm]	0.42		

The 3 mL L^–1^ solution of *L. calcareum* was prepared by dissolving 15 g of the commercial product Litho Micron 3000^®^ from the company Algadermis in 5 L of water. According to the manufacturer, the product is composed of 80% *Lithothamnium* seaweed and 20% calcium and magnesium silicate. The guaranteed levels of the product are 27% calcium, 1.8% magnesium, 2% silicon and 3% moisture. Its physical nature is powder. The chemical composition of nutrients and humic substances of *L. calcareum* used in this study is detailed in **Table 2**, adapted from the work of Ramos et al. (2023).

**Table 2:** Result of the chemical analysis of nutrients and humic substances of the product LT Supra^®^, from the former company Supramar (currently called Algadermis).

Parameter*	Unit	LT supra^®^
Nitrogen	[%]	0.06
Phosphor		0.09
Potassium		0.06
Calcium		31.19
Magnesium		2.06
Sulfur		0.29
Boron	[mg kg ^–1^]	48.06
Copper		0.97
Iron		14,765.56
Manganese		481.89
Zinc		10.5
Sodium		8,084.48
Fulvic acid	[%]	9.31
Humic acid		0.93

The product LT Supra^®^ also changed its name and is currently marketed as Litho Micron 3000^®^, maintaining the same formulation.* The product analyses were performed at the commercial laboratory ABCLab of the ABC Foundation from samples of commercial products, using the following methodology: EPA 6010/3051 (Brazil 2017), AOAC - Official Method 993.13 - Nitrogen (Total) in Fertilizers (Tate 1994).

Furthermore, the study by Ramos et al. (2023) indicates that the product based on L. calcareum has a concentration of 1,400 mg kg^−1^ of free amino acids (0.15%). Glycine and tryptophan stood out, both with 400 mg kg^−1^, in addition to aspartic acid (200 mg kg^−1^), alanine (200 mg kg^−1^), proline (100 mg kg^−1^), valine (100 mg kg^−1^) and glutamic acid (0.01 mg kg^−1^).

### 2.2 Water deficit imposition and experimental design

The recurrent water deficit experiment comprised three cycles, each including a dehydration phase (suspension of irrigation for five days) followed by a rehydration phase (resumption of irrigation for three days), called DRY1, DRY2, DRY3, and REC1, REC2, and REC3, respectively (Cerri Neto et al., 2023). With the exception of the control, which was not subjected to water deficit.

**Figure 1:**
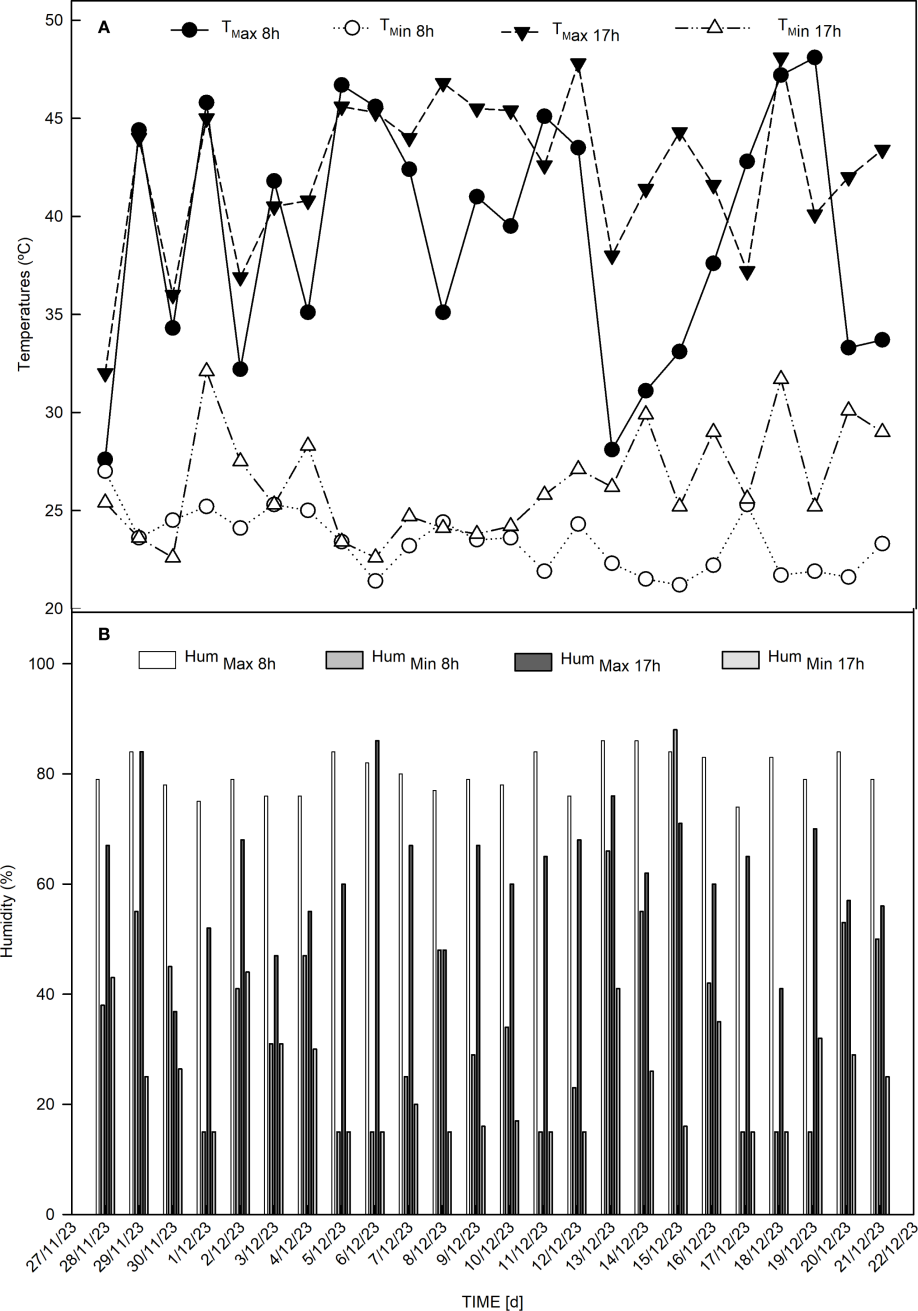
Climatic data. **(A)** Maximum temperature at 8am (T_max 8h_) [°C], minimum temperature at 8am (T_min 8h_) [°C], maximum temperature at 5pm (T_max 17h_) [°C] and minimum temperature at 5pm (T_min 17h_) [°C]. **(B)** Maximum humidity at 8am (Hum _max 8h_) [%], minimum humidity at 8am (Hum _max 8h_) [%], maximum humidity at 5pm (Hum _max 17h_) [%], minimum humidity at 5pm (Hum _max 17h_) [%].

Before each dehydration phase, the solutions corresponding to the treatments were applied. The experiment was conducted in a randomized block design, with four blocks, each containing 15 plants per plot. During the experiment, the temperature and relative humidity inside the greenhouse were measured using a digital thermometer-hygrometer, model Incoterm -7666.02.0.00. Readings were taken twice, at 8:00 am and 5:00 pm (**Figure 1**).

### 2.3 Water potential

Leaf water potential (Ψ_Leaf_) assessments were performed at the end of water deficit (DRY1, DRY2, and DRY3) and recovery (REC1, REC2, and REC3) cycles, with one plant per plot evaluated. Measurements were made at 5:00 AM using a Scholander pressure chamber (Model 1000, PMS Instrument Co., Albany, OR, USA) on healthy, fully expanded leaves collected from the middle third of the plants.

### 2.4 Vegetative growth

Plant development was assessed at the end of the third cycle of water deficit and rehydration, using ten plants per plot. Shoot development was assessed by leaf area (LA), stem diameter (SD), leaf dry mass (LDM), stem dry mass (SDM), total dry mass (TDM), shoot dry mass (DMAP) and specific stem length (SSL). Root development was measured by measuring the specific root length (SRL).

Leaf area was measured in cm^2^ LA, using the Scanner Area Meter LI-3100C benchtop device. SD was determined in the collar region with a precision digital parking meter, with values expressed in millimeters (mm). The allocation of leaf and stem dry mass, expressed in grams (g), was obtained by weighing the fractionated organs on a precision analytical balance, after drying in an oven with forced air circulation, at 65 °C, until constant weight.

TDM was calculated by the sum of the dry mass of the leaf, stem and root, while DMAP was obtained by the sum of the dry mass of the leaf and stem, both expressed in g. SSL was obtained by dividing the stem length by the dry mass, with the result expressed in cm g^–1^, as per (Poorter et al., 2011). SRL was calculated by dividing the root length by the dry mass, with the value also expressed in cm g^–1^, as per (Kramer-Walter et al., 2016).

### 2.5 Chlorophyll a fluorescence

Chlorophyll a fluorescence assessments were performed at the end of the water deficit (DRY1, DRY2, and DRY3) and recovery (REC1, REC2, and REC3) cycles, in two plants per plot, using a portable Pocket-PEA fluorometer (Hansatech, United Kingdom), as recommended by Strasser et al. (2004). For each plant, two leaves were adapted to the dark for 30 min, using specific clips, allowing complete oxidation of the photosystem. Then, a saturating light flash of 3,000 μmol photons m^–2^ s^–1^ was emitted, lasting 1 s. From the transient fluorescence OJIP, the parameters defined by the JIP test were calculated. The normalization and interpretation of the measured and calculated parameters followed the criteria of Strasser and Strasser (1995).

### 2.6 Photosynthetic pigments

The evaluations of chlorophyll *a*, *b* and total levels were performed at the end of the water deficit (DRY1, DRY2, and DRY3) and recovery (REC1, REC2, and REC3) cycles, in two plants per plot, using the electronic chlorophyll meter clorofiLOG, model CFL1030, from Falker. For each plant, two readings were performed.

### 2.7 Carbohydrate allocation

Carbohydrate allocation was assessed by quantifying reducing sugars (RS) and total soluble sugars (TSS) in the leaves, stems, and roots of ten plants per plot. Although the plants underwent three cycles of water deficit (DRY1, DRY2, and DRY3) and rehydration (REC1, REC2, and REC3), carbohydrate quantification was performed only at the end of the third cycle, after the rehydration phase. The dried plant organs were ground in a STAR FT-50 mill and stored in a freezer at –18°C.

Sugar extraction followed the method of Zanandrea et al. (2010). 0.2 g of the dried sample was homogenized in 5 mL of 0.1 M potassium phosphate buffer (pH 7.0), incubated in a water bath at 40°C for 30 min, and centrifuged using a NI 1811-A model at 5,000 rpm for 20 min. The supernatant was collected, and the precipitate was resuspended twice in 5 mL of the same potassium phosphate buffer. The combined supernatants were frozen for RS and TSS quantification, and the precipitate was frozen for starch extraction. The protocol used for RS quantification was the dinitrosalicylic acid method of Miller (1959), while TSS quantification followed a modified anthrone method of Yemm and Willis (1954), using 2 mL of 0.1% anthrone solution in 93.33% sulfuric acid, plus 1 mL of the plant extract, placed in a water bath at 100 °C for 3 min.

### 2.8 Statistical analysis

For variables such as water potential, chlorophyll a fluorescence, photosynthetic pigments, carbohydrate allocation and vegetative growth, analysis of variance (ANOVA) was performed. The means were compared by Tukey’s test at 5% probability (p < 0.05), using the SISVAR software version 5.8 (Ferreira, 2011).

## 3 Results

### 3.1 Water potential

**Figure 2:**
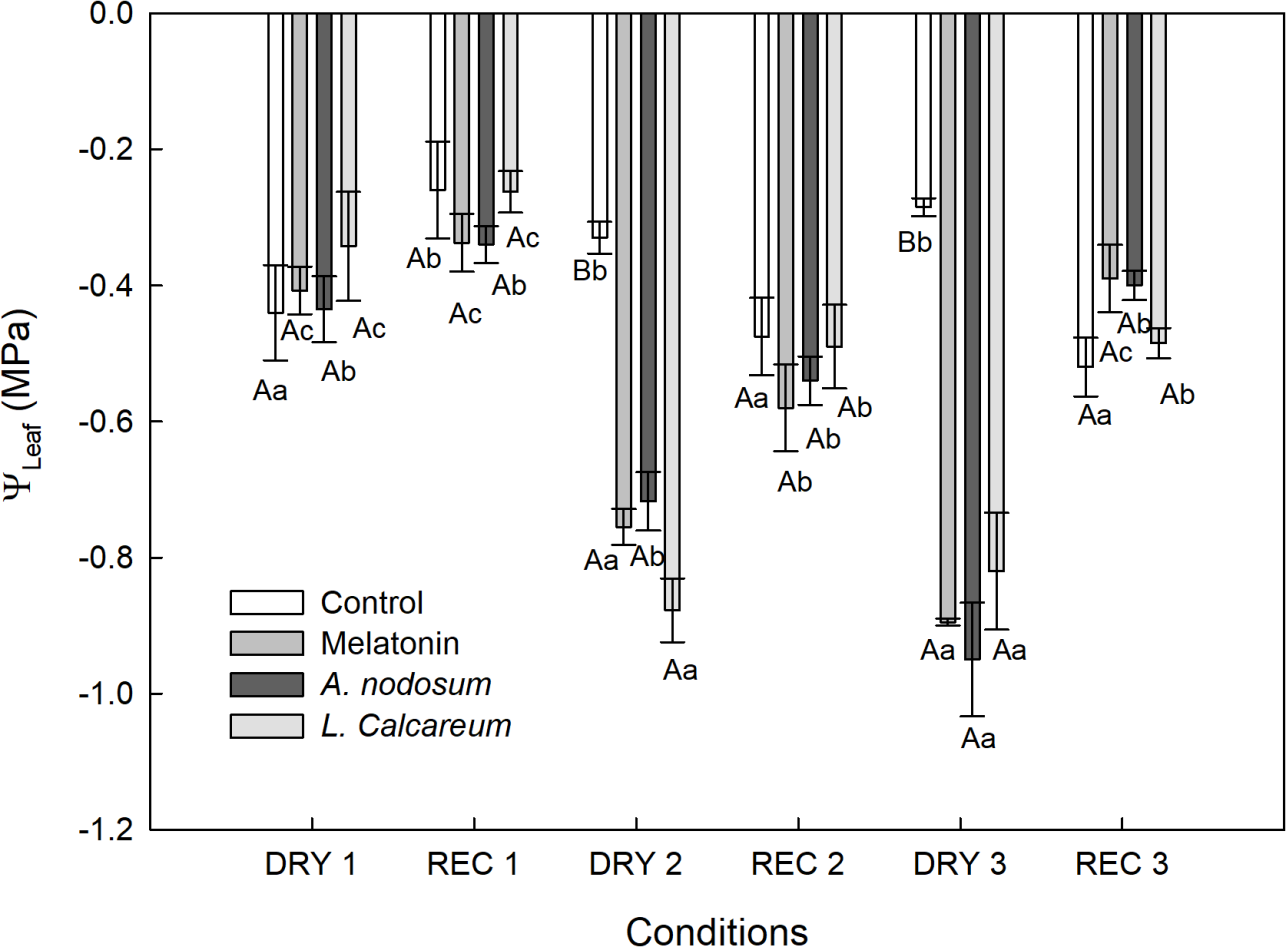
Effect of treatments with melatonin, *A. nodosum*, *L. calcareum* on leaf water potential (Ψ Leaf), measured in the morning of papaya (*Carica papaya* L.) ‘Aliança’ subjected to three cycles of water deficit (DRY1, 2 and 3) and recovery (REC1, 2 and 3). Means followed by the same letter do not differ from each other by the Scott-knott test (p<0.05). The bar corresponds to the standard error of four replicates containing one plant per plot. Capital letters are used to compare the effects of treatments within a given cycle, while lowercase letters compare the effect of treatments across all cycles.

In the first cycle of water deficit and recovery (DRY1 and REC1), all treatments presented similar leaf water potential (Ψ_Leaf_) (**Figure 2**), demonstrating that the plants were not under water deficit. However, in the second and third water deficit (DRY2 and DRY3), the treatments with melatonin, *Ascophyllum nodosum* and *Lithothamnium calcareum* presented Ψ_Leaf_ values statistically lower than the control and DRY1 (**Figure 2**), evidencing the presence of a more expressive water deficit. In the second and third rehydration (REC2 and REC3), the plants treated with melatonin, *A. nodosum* and *L. calcareum* equaled the Ψ_Leaf_ to the control (**Figure 2**).

Melatonin treatment showed higher Ψ_Leaf_ in DRY2 and DRY3 compared to DRY1 (**Figure 2**). Furthermore, it was observed that after the third drought and recovery cycle DRY3 and REC3, Ψ_Leaf_ showed an increase of 56.18%, going from -0.89 MPa to -0.39 MPa. This increase indicates a significant recovery in water potential, reflecting a lower water tension in the plant (**Figure 2**). In addition to the third cycle having shown an increase of 56.18% in Ψ_Leaf_, its recovery was faster compared to the second cycle DRY2 and REC2. In the second cycle, Ψ_Leaf_ increased by 22.67%, going from -0.75 MPa to -0.58 MPa after rehydration, evidencing a lower recovery compared to the third cycle.

In the treatment with *A. nodosum*, Ψ_Leaf_ was more expressive in DRY3. An increase in Ψ_Leaf_ of 57.89% was observed after REC3 (**Figure 2**). The treatment with *L. calcareum* showed a behavior similar to the melatonin treatment, with higher Ψ_Leaf_ values in DRY2 and DRY3 (**Figure 2**). The second DRY2 and REC2 cycle showed an increase of 43.68% in Ψ_Leaf_, ranging from -0.87 MPa to -0.49 MPa after rehydration. In the third cycle (DRY3 and REC3), the increase was 41.46%, from -0.82 MPa to -0.48 MPa. These results indicate that the recovery of Ψ_Leaf_ was slightly faster in the second cycle compared to the third (**Figure 2**).

### 3.2 Vegetative growth

There was no significant interaction between biostimulants and water deficit cycles; however, there were significant differences between the biostimulants tested. Leaf area (LA), stem diameter (SD), leaf dry mass (LDM), stem dry mass (SDM), total dry mass (TDM) and shoot dry mass (DMAP) showed significant reductions in all treatments with biostimulants when compared to the control, which was not subjected to water deficit (**Table 3**).

**Table 3:** Effect of treatments with melatonin, *A. nodosum*, *L. calcareum* on growth parameters of papaya (*Carica papaya* L.) ‘Aliança’ plants after three cycles of water deficit (DRY1, 2 and 3) and recovery (REC1, 2 and 3).

Treatments	LA [cm^2^]	SD [mm]	DLM [g]	DSM [g]
Control	1,876.51 ± 125.23 a	25.46 ± 0.16 a	4.56 ± 0.24 a	17.22 ± 1.27 a
Melatonin	1,223.43 ± 103.76 b	23.07 ± 0.48 b	2.88 ± 0.27 b	12.24 ± 0.82 b
*A. nodosum*	1,239.04 ± 112.68 b	23.06 ± 0.26 b	3.42 ± 0.28 b	13.63 ± 0.38 b
*L. calcareum*	1,190.84 ± 27.91 b	23.30 ± 0.49 b	3.11 ± 0.10 b	14.19 ± 0.57 b
Treatments	TDM [g]	DMAP [g]	SSL [cm g-1]	SRL [cm g-1]
Control	29.88 ± 1.86 a	21.79 ± 1.32 a	0.06 ± 0.001 b	0.07 ± 0.01 b
Melatonin	21.36 ± 1.09 b	15.12 ± 0.88 b	0.09 ± 0.001 a	0.10 ± 0.01 a
*A. nodosum*	24.51 ± 0.97 b	17.06 ± 0.24 b	0.07 ± 0.001 b	0.08 ± 0.01 b
*L. calcareum*	24.73 ± 0.78 b	17.30 ± 0.57 b	0.07 ± 0.001 b	0.08 ± 0.01 b

Values are means ± SE, n = 4. Means followed by the same letter do not differ from each other by the Scott-knott test (p < 0.05). LA, Leaf area; SD, stem diameter; DLM, leaf dry mass; DSM, stem dry mass; TDM, total dry mass; DMAP, shoot dry mass; SSL, specific stem length; SRL, specific root length.

However, the variables of specific stem length (SSL) and specific root length (SRL) showed a distinct behavior. Plants treated with melatonin presented higher values ​​for these variables. On the other hand, *A. nodosum* and *L. calcareum* did not differ from the control in these variables, showing less efficiency in promoting structural adjustments to stress (**Table 3**).

### 3.3 Chlorophyll a fluorescence

**Figure 3:**
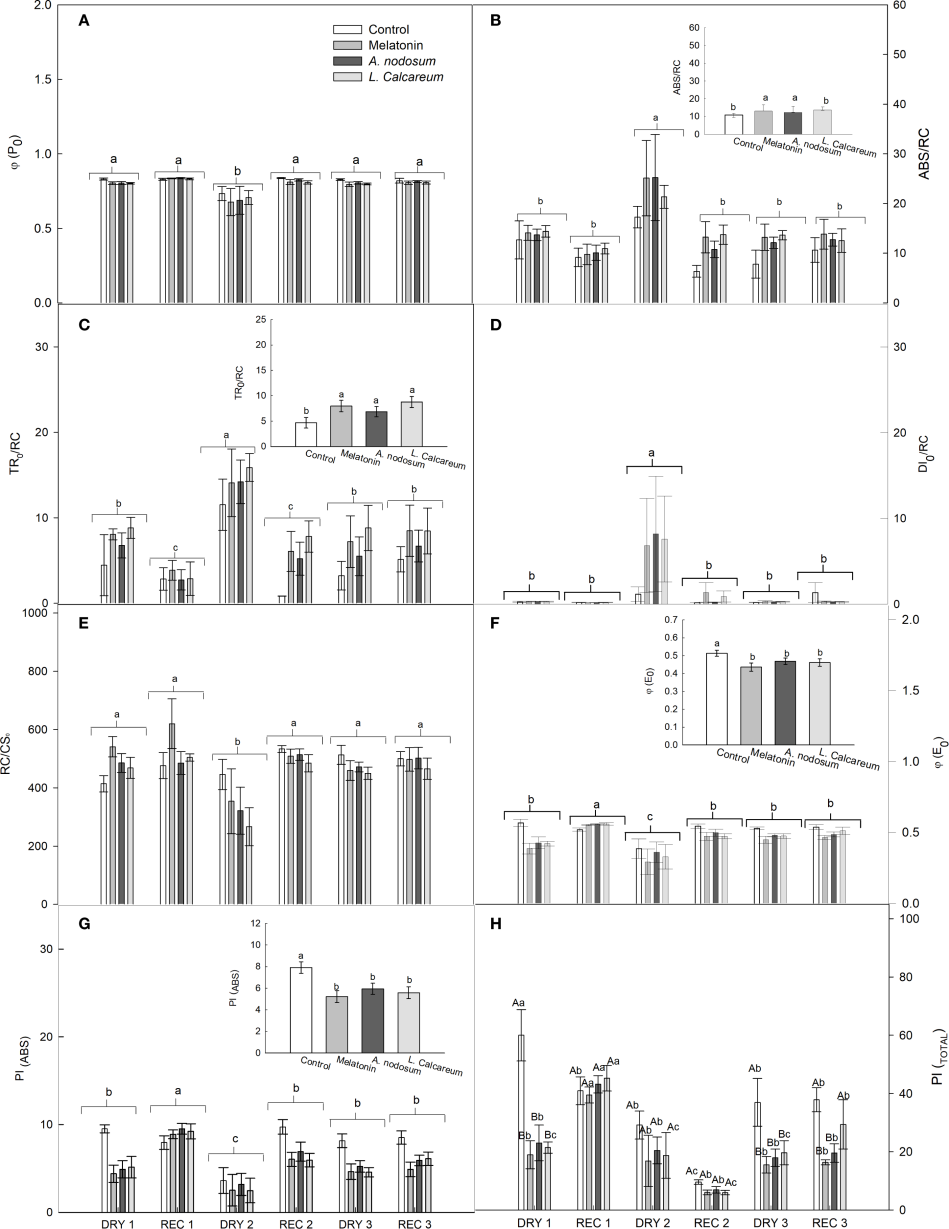
Effect of treatments with melatonin, *A. nodosum*, *L. calcareum* on photochemical parameters derived from the JIP test analysis of papaya (*Carica papaya* L.) ‘Aliança’ plants subjected to three cycles of water deficit (DRY1, 2 and 3) and recovery (REC1, 2 and 3). The bar corresponds to the standard error of four replicates containing the mean of two plants per plot. Means followed by the same letter do not differ from each other by the Scott-knott test (p<0.05). Capital letters are used to compare the effects of treatments within a given cycle, while lowercase letters compare the effect of treatments across all cycles. **(A)** Maximum photochemical quantum yield (φP_0_), **(B)** energy absorption flux per reaction center (ABS/RC), **(C)** captured energy flux per reaction center (TR_0_/RC), **(D)** dissipated energy flux per reaction center (DI_0_/RC), **(E)** number of active PSII reaction centers per cross sections (RC/CS_0_), **(F)** electron transfer quantum yield from QA– to the electron transport chain beyond QA– (φE_0_), **(G)** performance index for energy conservation of photons absorbed by PSII for the reduction of intersystem electron acceptors (PI_(ABS)_), **(H)** performance index for energy conservation of photons absorbed by PSII for the reduction of PSI final acceptors (PI_(Total)_).

For the variables in which no significant effects of interactions between treatments and water deficit and rehydration cycles were observed, the analysis was performed independently. In the maximum photochemical quantum yield (φP_0_), a significant difference was observed in the water deficit and rehydration cycles, with DRY2 presenting the lowest value in φP_0_ (**Figure 3A**). For the energy absorption flux per reaction center (ABS/RC), the treatments based on melatonin and *A. nodosum* presented significantly higher values compared to the control, evidencing greater energy absorption (**Figure 3B**). In the water deficit cycles, an increase in ABS/RC was observed in DRY2, while the other water deficit and rehydration cycles presented significantly lower values (**Figure 3B**).

For the energy flux captured per reaction center (TR_0_/RC), the treatments based on melatonin, *A. nodosum* and *L. calcareum* presented significantly higher values compared to the control (**Figure 3C**). Between the water deficit and rehydration cycles, DRY2 presented an increase in the TR_0_/RC value, while REC1 and REC2 presented lower values (**Figure 3C**).

A higher energy flux dissipated per reaction center (DI_0_/RC) was observed in DRY2, while the other water deficit and rehydration conditions presented significantly lower values, with no significant differences between them. No significant differences were observed between the treatments for this variable (**Figure 3D**). Likewise, no significant differences were observed between the treatments for the number of active PSII reaction centers per cross section (RC/CS_0_). However, under water conditions, DRY2 presented the lowest number of active reaction centers, while the other treatments demonstrated higher and statistically similar values (**Figure 3E**).

In the quantum efficiency of electron transfer from QA – to the electron transport chain beyond QA– (φE_0_) and in the performance index for energy conservation of photons absorbed by PSII for the reduction of intersystemic electron acceptors PI_(ABS)_, the control presented higher values. While the treatments reduced this efficiency, without significant differences between them (**Figures 3F, G**). Between the water deficit and rehydration cycles, REC1 obtained higher values of φE_0_ and PI_(ABS)_, while DRY2 presented reduced values (**Figures 3F, G**).

There was a significant interaction between the treatments and the water deficit and rehydration cycles, in the performance index for energy conservation of photons absorbed by PSII for the reduction of the final acceptors of PSI (PI_(Total)_). In DRY1, the treatments presented values lower than the control; however, in REC1, the PI_(Total)_ values in the treatments were statistically equal to those of the control (**Figure 3H**). In DRY2 and REC2, the PI_(Total)_ values remained similar to the control, indicating a more uniform recovery during this period (**Figure 3H**). DRY3 had the same behavior as DRY1; however, in REC3, only the biostimulant based on *L. calcareum* was able to maintain PI_(Total)_ values statistically equal to the control (**Figure 3H**). Melatonin and *A. nodosum* did not recover the PI_(Total)_ values to the same level as the control, highlighting differences in performance between the treatments evaluated (**Figure 3H**).

Melatonin and *A. nodosum* showed a significantly higher PI_(Total)_ in REC1 compared to the other water conditions, with an increase of 108.23% and 87.45% for melatonin and *A. nodosum* respectively, indicating a significant recovery in this cycle. However, melatonin and *A. nodosum* did not promote the recovery of PI_(Total)_ in subsequent cycles. In *L. Calcareum*, PI_(Total)_ was more significant in REC1. The first cycle (DRY1 and REC1) showed an increase of 111.32% in PI_(Total)_, ranging from 21.4100 to 45.2400 after rehydration. There was no recovery of PI_(Total)_ in the second drought cycle, and the third cycle showed a lower recovery than the first cycle, of only 49.4%.

### 3.4 Photosynthetic pigments

**Figure 4:**
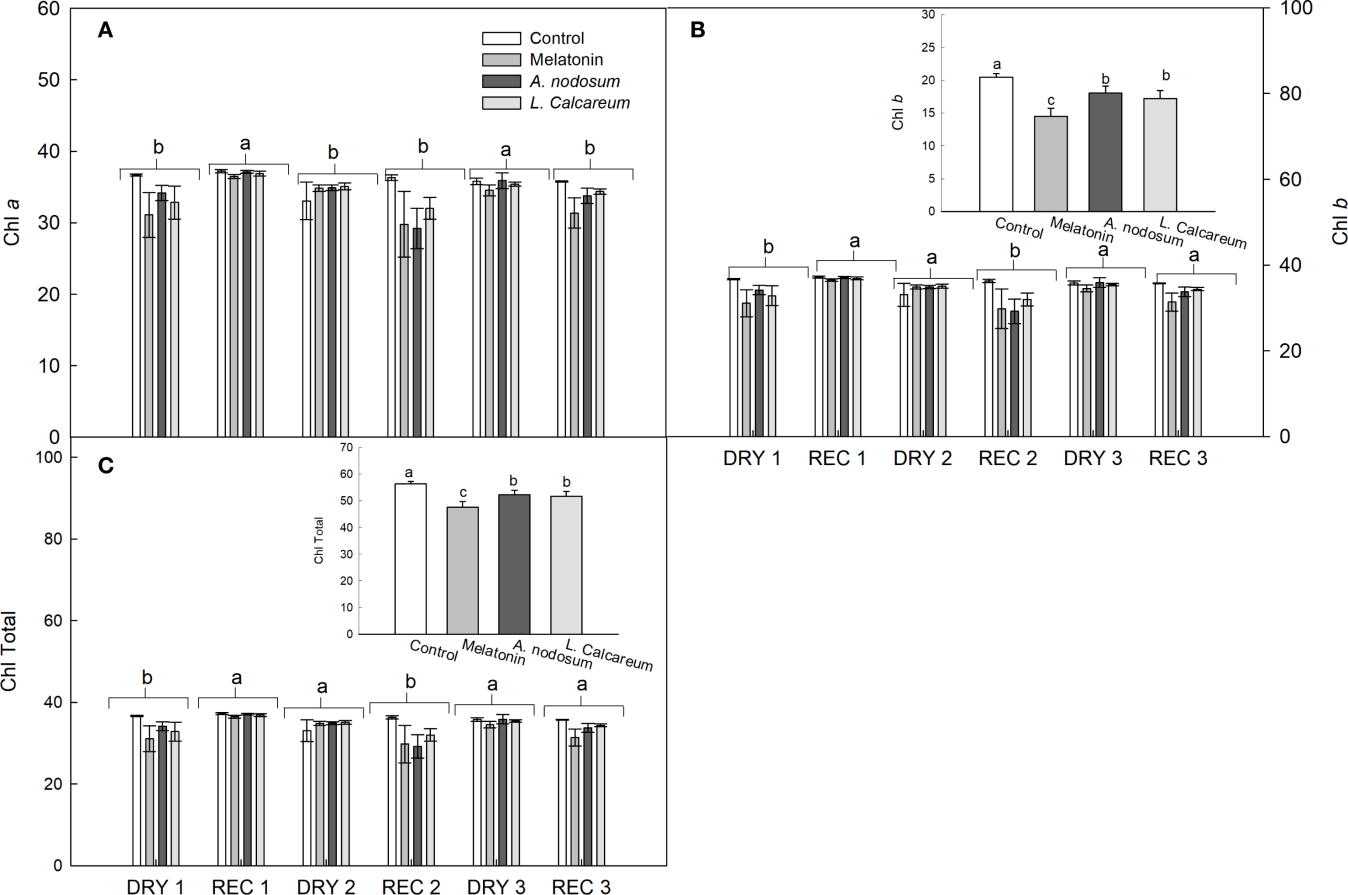
Effect of treatments with melatonin, *A. nodosum*, *L. calcareum* on the photosynthetic pigment content of papaya (*Carica papaya* L.) ‘Aliança’ subjected to three cycles of water deficit (DRY1, 2 and 3) and recovery (REC1, 2 and 3). The bar corresponds to the standard error of four replicates containing the mean of two plants per plot. Means followed by the same letter do not differ from each other by the Scott-knott test (p<0.05). **(A)** chlorophyll a (Chl *a*), **(B)** chlorophyll b (Chl *b*), **(C)** chlorophylls total (Chl Total).

For the variables in which no significant effects of interactions between treatments and water deficit and rehydration cycles were observed, the analysis was performed independently. Regarding the chlorophyll a (Chl *a*) content, a significant difference was observed between the water deficit and rehydration cycles. REC1 and DRY3 presented higher values compared to the other water conditions (**Figure 4A**).

Regarding the chlorophyll b (Chl *b*) and total chlorophyll (Chl total) contents, a significant difference was observed in the treatments and in the water deficit and rehydration cycles (**Figures 4B, C**). After the first water deficit cycle, there was a recovery of 36.44% and 17.82% of the Chl *b* and Chl total contents, respectively (**Figures 4B, C**). In DRY2, the plants maintained the Chl *b* and Chl total contents statistically similar to REC1 (**Figures 4B, C**). However, after being rehydrated (REC2), the Chl *b* and Chl total contents decreased when compared to DRY2 (**Figures 4B, C**).

In the third cycle, the Chl *b* and Chl total contents increased, remaining statistically similar to the values of REC1 (**Figures 4B, C**). The treatments reduced the Chl *b* and Chl total contents when compared to the control. However, the values of Chl *b* and Chl total contents in the treatments with *A. nodosum* and *L. calcareum* were higher than those observed in the treatment with melatonin.

### 3.5 Carbohydrate allocation

**Figure 5:**
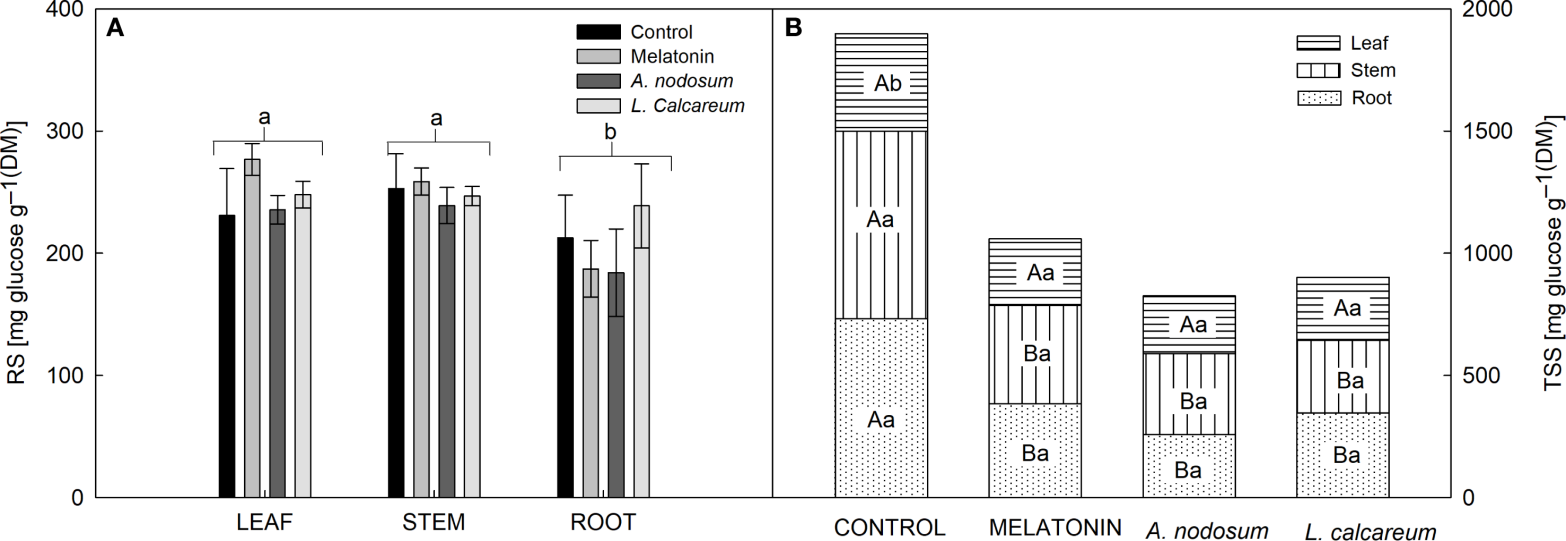
Effect of treatments with melatonin, *A. nodosum*, *L. calcareum* on the allocation of carbohydrates in papaya (*Carica papaya* L.) ‘Aliança’ plants after three cycles of water deficit (DRY1, 2 and 3) and recovery (REC1, 2 and 3). The bar corresponds to the standard error of four replicates containing the mean of ten plants per plot. Means followed by the same letter do not differ from each other by the Scott-knott test (p<0.05). In the interaction, capital letters compare the TSS content of the same organ between treatments, while lowercase letters compare the TSS content of the same treatment between organs. **(A)** reducing sugar (RS), **(B)** total soluble sugar (TSS).

No significant effects were observed between treatments and vegetative organs regarding reducing sugar (RS) levels. However, regardless of the treatments, there was a greater accumulation of RS in the leaf and stem (**Figure 5A**). However, interactions between treatments and vegetative organs were observed regarding total soluble sugar (TSS) levels. The treatments melatonin, *A. nodosum*, and *L. calcareum* reduced TSS levels in the stem and root compared to the control (**Figure 5B**). However, there was no statistical difference between the vegetative organs in the treatments (**Figure 5B**).

## 4 Discussion

Melatonin treated plants showed a reduction in Ψ_Leaf_ in DRY2 and DRY3; however, after water return, Ψ_Leaf_ increased in these cycles (**Figure 2**). Ψ_Leaf_ recovery was more significant in the third cycle, possibly due to the cumulative effect of melatonin over periods of water deficit. This effect may be related to melatonin’s ability to stimulate the production of osmotic solutes, such as carbohydrates, which play a central role in regulating osmotic potential, contributing to the preservation of relative water content and cellular functionality (Goñi et al., 2018; Frioni et al., 2021; El-Yazied et al., 2022).

Melatonin treated plants showed an increase in specific stem length and specific root length (**Table 3**) and a decrease in total soluble sugars in the stem and root (**Figure 5B**). These results suggest that total soluble sugars were mobilized as an energy source to sustain the growth of these organs, reflecting metabolic adjustments to water stress. Root changes are considered essential adaptive mechanisms for plants to withstand stressful conditions (Zulfiqar et al., 2020), being fundamental for optimizing water and nutrient uptake in environments with low water availability (Cerri Neto et al., 2023). The result of the current study is corroborated by the research of Hossain et al. (2020), who reported that the application of exogenous melatonin improved the root length of wheat *Fagopyrum tataricum* (L.) Gaertn under water deficit.

Water deficit can also compromise the ability of *Carica papaya* stems to maintain adequate turgor pressure, essential for supporting the weight of the fruit, which can lead to plant bending (Campostrini et al., 2018). However, exogenous melatonin application can help mitigate these effects. A study by Zhao et al. (2022) demonstrated that melatonin application to *Paeonia lactiflora* Pall. plants resulted in increased stem length, promoting greater plant resistance. Thus, the improvement in Ψ_Leaf_ recovery, as observed in the second and third recovery cycles in this study, may be associated not only with the osmotic regulation promoted by melatonin, but also with the balanced growth between stem and root, allowing a more effective response to water return and contributing to the structural stability of the plant.

It is noted that ‘Aliança’ papaya plants treated with *A. nodosum* showed a reduction in Ψ_Leaf_ in DRY3, followed by an increase in this parameter after rehydration (**Figure 2**). This result may be associated with the chemical composition of *A. nodosum* based products, which contain a wide range of inorganic components, such as K, Ca, Mg, and Zn (Rayirath et al., 2009; Battacharyya et al., 2015). These nutrients promote defense responses and osmotic adjustment (Ahmed et al., 2024). Studies with grasses have shown that the application of *A. nodosum* extracts conferred greater heat resistance, an effect attributed to increased K uptake by plants (Ervin et al., 2004; Zhang and Ervin, 2008). This mechanism reinforces the importance of K in stomatal regulation, also highlighted by Van Oosten et al. (2017), who emphasized its accumulation as a crucial step in protecting against osmotic stress.

Spann and Little (2011) reported that orange (*Citrus sinensis* L.) plants subjected to water stress and treated with commercial *A. nodosum* extracts showed improved water status and greater water use efficiency. These findings corroborate the improvement in Ψ_Leaf_ recovery observed in the present study, suggesting that *A. nodosum* based biostimulants may contribute to the resilience of ‘Aliança’ papaya under water deficit conditions, favoring the maintenance of water potential and cellular homeostasis.

Furthermore, the organic carbon composition of Baltiko^®^, which may be associated with the presence of a variety of active substances derived from *A. nodosum* extract, such as fucoidan, laminarin, alginic acid, mannitol, betaines, oligosaccharides, and sterols (Shukla et al., 2019; Pereira et al., 2020; Ahmed et al., 2024), may be associated with the stimulation of plant defense responses and are described in the literature as responsible for biostimulant effects under stressful conditions (Shukla et al., 2019; Ahmed et al., 2024). Compounds such as glycine betaine and sterols act as osmolytes, helping to maintain osmotic potential and preserve cell turgor, which prevents structural damage under conditions of low water availability in plants (Shukla et al., 2019).

Likewise, melatonin and *A. nodosum*, *Lithothamnion* sp. reduced Ψ_Leaf_ values in DRY2 and DRY3, however, after rehydration the Ψ_Leaf_ values increased (**Figure 2**). The biostimulant effects of *Lithothamnion* sp. and calcareous algae, promoting better growth and development, have been reported in several plant species (Bamaniya et al., 2024; Singh et al., 2025), such as jatropha (Evangelista et al., 2016), Arabica coffee (Rodriguez et al., 2017), tomato (Amatussi et al., 2020), beans (Marques et al., 2023), cucumber (Kakbra, 2024), ornamental plants (Yücedağ and Çiçek, 2024), potato (Amatussi et al., 2025), melon (Marques et al., 2025), and papaya (Ramos et al., 2025).

*L. calcareum* may aid in osmotic regulation due to its high calcium (Ca^2+^) and magnesium (Mg^2+^) content (Silva et al., 2023). These cations are essential for the stability of cell membranes and the activation of several metabolic enzymes, providing greater resistance to water stress and contributing to cellular homeostasis (Pirayesh et al., 2021; Ishfaq et al., 2022). Furthermore, commercial products based on *L. calcareum* contain proline (Ramos et al., 2023). Proline, in addition to protecting plant metabolism against damage caused by reactive oxygen species, also plays a crucial role in quenching singlet oxygen and scavenging hydroxyl radicals (ROS) (Spann and Little, 2011). The presence of humic acid in the *L. calcareum* product aids in the activation of antioxidant enzymes, increasing ROS scavenging (Spann and Little, 2011).

For the φP_0_ values in DRY2, a reduction was observed in DRY2 (**Figure 3A**), indicating the occurrence of intense damage in the QA reduction capacity, due to the impairment in the excitation energy transfer capacity of PSII (Marutani et al., 2012). Consequently, φE_0_ was compromised (**Figure 3F**), the reduction in φE_0_ is a result of the failure of the electron transport efficiency (Jiang et al., 2008). Although there were increases in the ABS/RC absorption components and in the TR_0_/RC energy capture component observed in DRY2 (**Figures 3B, C**), they did not result in an increase in φP_0_ efficiency and φE_0_ yield. Melatonin and *A. nodosum* application also increased the absorption and capture components (ABS/RC and TR_0_/RC) (**Figures 3B, C**); however, it did not increase the photosynthetic index PI_(ABS)_ (**Figure 3G**).

Stressed plants increase the inactivation rates of reaction centers (Souza et al., 2022). Indeed, a reduction in active RC/CS_0_ reaction centers was observed (**Figure 3E**). The reduction in RC/CS_0_ refers to the lower density of reaction centers capable of reducing QA, forcing an energy overload on the reaction centers that remained active (Zhang et al., 2018). Reaction center inactivation increases under water deficit, so that all light energy reaching the active reaction centers tends to cause a high level of excitation in LHCII and PSII, reflecting the high absorption and capture of this energy (Dimitrova et al., 2020). However, not all of the received excitation energy is able to be transported through the transport chain, as electron flow through the oxygen-evolving complex (OEC) is limited by water stress, also resulting in high energy dissipation (Chen et al., 2016).

The photosynthetic performance indices PI_(ABS)_ and PI_(Total)_ are multiparametric, as they involve other parameters, such as the absorption component (ABS), the capture component (TR), and the electron transport component ET (Strasser et al., 2004). PI(ABS) is considered the sensitive index for water stress (Strasser et al., 2004; Yusuf et al., 2010; Kalaji et al., 2016). The reduction of PI_(ABS)_ in DRY2 and between treatments (**Figure 3G**) suggests a decrease in overall photosynthetic performance associated with reduced electron transport capacity (Kalaji et al., 2018). Furthermore, the reduction in PI_(Total)_ during DRY1 and 3, except for *L. calcareum*, is associated with a further slowing of the reduction of PSI end acceptors (Strasser et al., 2010). Therefore, the decrease in PI_(ABS)_ and PI_(Total)_ in ‘Aliança’ papaya plants, as observed during the recurring drought cycle, is associated with the stress conditions to which the plants were subjected, which may cause direct and indirect damage to the photosynthetic process of the plants (Schock et al., 2014). However, the ‘Aliança’ papaya plants treated with melatonin, *A. nodosum* and *L. calcareum*, showed significant improvements in PI_(ABS)_ and PI_(Total)_ values during REC1, when compared to the same drought cycle. This suggests that the plants under these conditions had a greater capacity to utilize light for photosynthesis, indicating an improvement in the conversion of light energy into energy, favoring the recovery of their functions.

We highlight that foliar applications of melatonin, *A. nodosum*, and *L. calcareum* resulted in significant changes in leaf water potential, chlorophyll a fluorescence, photosynthetic pigments, carbohydrate allocation, and vegetative growth of ‘Aliança’ papaya seedlings. These results suggest that these substances aid in adjusting the plants’ morphophysiological mechanisms, playing an essential role in the seedlings’ ability to tolerate recurring cycles of water deficit. In practice, the use of these substances can assist farmers in cases of abiotic stress generated by water deficit, improving the plant’s photosynthetic capacity and regulating its growth under adverse environmental conditions, ensuring crop survival. We also highlight that complementary studies with the inclusion of new characteristics analyzed, such as gene expression of marker genes and the quantification of hormones such as abscisic acid (ABA), which play a known role in response to drought, can be applied in future studies to better understand the response of papaya to water deficit.

## 5 Conclusions

Seaweed and melatonin had a positive effect on papaya seedlings, mitigating the negative effects of water stress, likely due to increased water retention through a decrease in leaf water potential, maintenance and recovery of photosynthetic functions, and contributing to vegetative growth. Chlorophyll a fluorescence demonstrates that during the second cycle of water deficit, there were significant reductions in maximum photochemical quantum yield (φP_0_) and an increase in the flux of energy dissipated per reaction center (DI_0_/RC), with inhibition of photosynthetic functions during this period.

However, seaweed and melatonin applications reduced total soluble sugar levels, and in the case of melatonin, this reduction was accompanied by a greater investment in the growth of specific stem length and specific root length, essential characteristics for adaptation to water stress.

Furthermore, although seaweed and melatonin did not promote significant gains in leaf area, stem diameter, leaf dry mass, stem dry mass, total dry mass, and shoot dry mass in papaya seedlings, the physiological adjustments observed in leaf water potential and photosynthetic functions indicate that these treatments may contribute to plant resilience under water stress conditions.

## Data Availability

The original contributions presented in the study are included in the article/supplementary material. Further inquiries can be directed to the corresponding author.
